# Discovering patterns and trends in customer service technologies patents using large language model

**DOI:** 10.1016/j.heliyon.2024.e34701

**Published:** 2024-07-19

**Authors:** Chaeyeon Kim, Juyong Lee

**Affiliations:** Department of Industrial and Systems Engineering, College of Engineering, Changwon National University, Changwondaehak-ro 20, Changwon-si, Gyeonsangnam-do, 51140, South Korea

**Keywords:** Customer service, Digital transformation, BERTopic, Cloud computing, Large language model

## Abstract

The definition of service has evolved from a focus on material value in manufacturing before the 2000s to a customer-centric value based on the significant growth of the service industry. Digital transformation has become essential for companies in the service industry due to the incorporation of digital technology through the Fourth Industrial Revolution and COVID-19. This study utilised Bidirectional Encoder Representations from Transformer (BERT) to analyse 3029 international patents related to the customer service industry and digital transformation registered between 2000 and 2022. Through topic modelling, this study identified 10 major topics in the customer service industry and analysed their yearly trends. Our findings show that as of 2022, the trend with the highest frequency is user-centric network service design, while cloud computing has experienced the steepest increase in the last five years. User-centric network services have been steadily developing since the inception of the Internet. Cloud computing is one of the key technologies being developed intensively in 2023 for the digital transformation of customer service. This study identifies time series trends of customer service industry patents and suggests the effectiveness of using BERTopic to predict future trends in technology.

## Highlights

• Propose a methodology identifying technical trends in digital transformation.
• Perform text mining on patents from the last 23 years of customer service patents.
• Cloud computing was identified as one of the key technologies in the CS industry.
• Large language models help with technology prediction and topic modelling.

## 1 Introduction

Before the 2000s, service referred to material value centred on manufacturing. However, in the 21st century, with the rapid development of technology and the growing size of the service industry market, the meaning of service has shifted towards customer-centric value. This change has affected the entire service industry, with increasing expectations of customer demands and an emphasis on convenience and corporate social responsibility (CSR). Since 2011, the Fourth Industrial Revolution has led to an increased influence of digital technology, including its integration into the service industry. The exponential growth of data has resulted in attempts to create new value, with companies promoting digital transformation as a new management strategy. The definition of digital transformation, according to the dictionary, is the process through which companies and organisations use digital technologies and tools to change their business models and processes [1]. According to the definition of digital transformation published by the Digital Initiative Group (shown in Table 1), companies define digital transformation differently based on their level of digital integration [2]. However, it is clear that digital transformation involves changing direction in response to the digital industry.

**Table 1:** Definitions of digital transformation.

Corporation	Definition
Bain & company	Making a difference by redefining the digital enterprise industry digitally and fundamentally reversing the laws of gaming
AT Kearney	Business activities to proactively respond to changes in the business environment triggered by new digital technologies such as mobile, cloud, big data, artificial intelligence, and the Internet of Things, and to dramatically increase the competitiveness of the current business or pursue new growth through new businesses
PWC	In corporation management, a set of processes that apply what digital consumers and ecosystems expect to business models and operations
Microsoft	To create new value for customers, embrace new ways to reform existing business models and combine people, data, and processes through intelligent systems
IBM	Companies integrate digital and physical elements to transform their business models and set new directions for the industry
IDC	Creating new business models, product services based on digital capabilities as customers and markets change, applying them to management, and making them sustainable
World Economic Forum	Change organization by leveraging business models that can improve digital technology and performance

The COVID-19 pandemic has led to the implementation of ‘social distancing’ policies, resulting in a shift towards non-face-to-face interactions in many aspects of our lives [3]. This has accelerated the digital transformation of the customer service industry, driven by the core technologies of the Fourth Industrial Revolution. This transformation aims to provide customers with more personalized experiences and greater convenience. As a result, digital transformation has become a necessity for most companies. To adapt to the growing significance of digital transformation, it is essential to identify the technologies associated with it in the customer service industry and analyse their evolution from various perspectives over time.

Topic modelling is a statistical technique used to estimate the topic of a document by determining the probability that each word in a set of documents belongs to a particular topic. It is employed in various fields to extract insights and gain information by identifying highly relevant words or topics from large amounts of text data. A study conducted in the agricultural field found that Latent Dirichlet Allocation (LDA) topic modelling was able to identify crop names and soil with 80 % accuracy in 3000 sentences extracted from agriculture-related sites [4]. A study conducted topic modelling using LDA on 2679 mountain livestock farming related literature published from 1980 to 2018 and suggested the need for a holistic research approach to the problems faced by livestock farming in mountainous regions [5].

In the field of manufacturing, Wang and Hsu used LDA method to identify potential technical topics in smart manufacturing and extracted 14 topics from 5521 patent literatures, among which, smart connection, manufacturing data analytics and power bed fusion additive manufacturing were identified as highly salient and valuable topics, which provided practical insights for technology development and R&D invest decisions [6]. Feng et al. conducted LDA-based topic modelling on 255 patent documents to generate 26 business topics, categorised into nine components of a business model canvas, and applied generative topographic mapping to present a systematic approach to identify business opportunities for manufacturers to develop new sustainable business models [7]. In the service provision industry, there is a study that analysed 1075 dental patients’ complaints collected as textual data using LDA method to identify 12 types of value co-destruction (VCD) and identified antecedents of VCD [8]. Ray et al. conducted topic modelling on 3570 articles to explore factors that influence the motivation to use e-learning services [9]. Putranto et al. conducted a topic modelling study using LDA on more than 50,000 samples collected from 510 hotels across Indonesia and found that the most common topics discussed in customer reviews were service, price/food, facilities, comfort, and location [10].

Similarly, in the medical field, a study using Structural Topic Modelling (STM) identified 11 useful patterns from a dataset of opioid drug reports, consisting of a total of 3069 unique reports [11]. In addition, Corti et al. collected a total of 2,458,929 tweets posted from 2019 to 2020 to analyse topics related to autism spectrum disorder through social media Twitter, and performed topic modelling using the NMF method [12]. In the field of finance, two studies have been conducted using LDA to perform topic modelling. The first study analysed 432 articles on blockchain technology to suggest future research trends on blockchain technology to suggest future research trends on blockchain trustworthiness [13]. The second study extracted topics from consumer complaints received by the Consumer Financial Protection Bureau (CFPB) to explore trends in consumer complaints over time [14]. Further, Aziz et al. leveraged LDA to extract 14 research themes from 5204 academic articles published between 1990 and 2018, with the aim pf providing a structured terrain for finance researches looking to integrate machine learning research approaches into their exploration of financial phenomena [15].

Perez-Segura et al. utilised a neural network based on the Bidirectional Encoder Representations from Transformer (BERT) architecture to analyse the abstracts of 1048 scientific papers [16]. Their findings revealed that research on climate change migration can be categorised into 15 distinct topics. In addition, Ferguson et al. employed the Short Text Topic Modelling (STTM) method to topic model a data set of 250,000 enterprise social network messages from 32 teams enrolled in a three-month intensive product design course [17]. This allowed them to identify communication patterns throughout the product design process.

Topic modelling also has been applied in the field of customer service. Most studies in this area used customer reviews of specific services as data for analysis. Hendry et al. employed LDA to identify customer intent, discover new intentions, and reconstruct existing ones to enhance a company's chatbot system [18]. Barravecchia et al. conducted a case study using STM for Digital Voice of Customer (DVOC) analysis on approximately 17,000 reviews from Yelp, Google, Trustpilot, Facebook, and the Play Store for 22 ride-sharing service providers from January 2010 to December 2019 [19]. Mishra analysed a dataset of 25,173 customer tweets related to an e-retailer [20]. LDA was used to output five themes of customer experience tweets related to online, providing insights into customer experience. Ibrahim and Wang collected tweets related to five major UK online retailers from Black Friday to Christmas and New Year sales [21]. The aim was to identify the main topics of customer interest in online retail brands shared among Twitter users. They then conducted topic modelling to derive insights into how well leading online retail brands are performing and how their products and services are perceived by customers. Ding et al. employed a structural topic model to extract service quality attributes from 2420 Airbnb reviews in Malaysia [22]. The study analysed the varying preferences of international Airbnb users and the changing patterns of the top six service quality attributes over five years. The findings indicate that the appearance of the property and communication with the host are increasingly important factors in users' stay experience.

While there have been studies on topic modelling using data such as messages, service reviews, and social media to improve customer service, there are no studies that analyse patent data to identify trends in related technologies in the field of customer service to the best of our knowledge. There is a study that use non-patent academic literature data to identify the interconnections between various Industry 4.0 technologies, and analyse publication trends and keyword changes over time [23]. A rare study of topic modelling using large language models such as BERTopic, which is used as an analytical tool in this study, is the study of Egger and Yu, who analysed Twitter data using four topic modelling techniques - LDA, NMF, Top2Vec and BERTopic – to bridge the evolving fields of computational science and empirical social research, and found that BERTopic generates new insights using an embedding approach in addition to topics [24]. Uncovska et al. compared a total of 17,588 German reviews of 15 German prescription-based mobile health (mHealth) apps and 50 non-prescription mHealth apps in Germany and conducted sentiment analysis and topic modelling using BERTopic to identify consumer interests and key factors for each app [25].

This study aims to identify trends and provide insights into patent data related to customer service using topic modelling method. Patent data can reveal trends in industries and technologies across various fields and are frequently used to identify relevant technologies in a given field at a particular time [26]. This study analyses the trend of topics over time in the customer service field by crawling patent data from January 1, 2000 to 2022 and performing topic modelling using BERT. This study is organised in the following sequence Section 2 provides a theoretical description and overview of the BERTopic model, followed by a description of the analysis process and data; Section 3 presents the results of the topic modelling and draws implications; Section 4 presents a summary of the findings, conclusions and limitations.

## 2 Methods and data

This study used BERTopic as a patent data topic modelling methodology. BERTopic is a transformer method for topic modelling using pre-trained language models called BERT, and its core is the use of BERT-based embeddings and class-based text frequency-inverse document frequency (c-TF-IDF) [27].

Transformer is an artificial intelligence language model announced by Google in 2017, and it has revolutionised the field of natural language processing by overcoming the problem of gradient descent, a shortcoming of recurrent neural networks, which have been the primary method of natural language processing [28]. Transformer has a structure consisting of an encoder and a decoder. The attentional function, which plays a key role in the Transformer architecture, calculates the importance of each word independently and comprehensively considers information about words in all positions, greatly improving the performance of natural language processing. Since the announcement of Transformer, other large language models such as BERT and GPT (Generative Pretrained Transformer) have emerged based on it. BERT was announced by Google in 2018 and is an implementation of the Transformer encoder only [29]. It acquires rich contextual information through pre-training on large-scale data, and by applying a bidirectional attachment mechanism in the encoder to consider contextual information in both directions, it has the characteristics of more sophisticated understanding of word interaction and contextual characteristics. Due to these features, BERT has shown excellent performance in various natural language processing tasks, and depending on the problem, fine-tuning or transfer learning has been shown to be effective in solving the problem, and has been studied in various language processing fields such as classification, machine reading, and machine translation [30].

The basic structure of BERT is an encoder stacked on top of a transformer, which has been used as a general-purpose model in natural language processing. It is characterised by the fact that various natural language processing tasks can be solved by a trained model with the same structure. The main advantage of BERT is that it can be fine-tuned with a small dataset based on pre-trained embeddings and then applied to other analyses with good performance [31]. Once the embeddings are created by training on a large amount of unlabelled data in the pre-training phase, the fine-tuning phase uses them to train on a small amount of labelled data to solve specific tasks.

Once the text data for topic modelling is collected, BERTopic goes through five steps to extract keywords that represent topics. First, BERT is used to perform embedding for each document. Each document is embedded in a high-dimensional space, and this process learns a meaningful representation by considering the words and context of each document.

Second, the Uniform Manifold Approximation (UMAP) is used to reduce the dimensionality of each document vector [32]. This step is crucial in making the data suitable for clustering and topic analysis. UMAP is preferred due to its ability to accurately preserve non-linear structures, making it ideal for visualising complex data structures while also preserving local structures. This approach facilitates the differentiation of topics by grouping similar documents and separating those with dissimilar topics.

Third, a clustering process is employed using Hierarchical Density-based Spatial Clustering of Applications with Noise (HDBSCAN) to identify dense clusters and define them as topics [33]. The hierarchical structure of HDBSCAN topic in BERTopic allows for easy comprehension of the relative relationship between each topic, by utilizing the hierarchical nature of HDBSCAN.

Fourthly, c-TF-IDF is used to model the importance of each word from a topic perspective. TF-IDF is a statistical method for representing words that assigns weights to each word in the document word matrix based on its importance [34]. The weight is calculated as the product of term frequency and inverse document frequency. C-TF-IDF is a method that calculates TF-IDF by grouping clusters and adjusting the weight by considering the distance between the centre of each cluster and the word in the general TF-IDF method. This method is effective in obtaining more meaningful information than the document word matrix because it considers the importance of words. However, it does not reflect the meaning of words as it only considers the number of occurrences of words. The formula below shows how TF-IDF is calculated.

$$c-\text{TF}-{IDF}_{i}=\frac{t_{i}}{w_{i}}\times \log\frac{m}{\sum_{j}^{n}t_{j}}$$

$$c-\text{TF}-{IDF}_{i}:c-TF-IDF\;weight\;for\;term\;i$$

$$t_{i}:\text{Term}\;\text{frequency}\;\text{of}\;i$$

$$w_{i}:weight\;of\;i$$

m:Totalnumberofdocuments

$$\sum_{j}^{n}t_{j}:\text{The}\;\text{sum}\;\text{of}\;\text{the}\;\text{frequency}\;\text{of}\;\text{all}\;\text{terms}$$

Finally, once the importance modelling of the words is complete, the topics are identified for each cluster and keywords representative of each topic are extracted. Various methodologies exist for topic modelling, including LDA [35], Latent Semantic Analysis (LSA) [36], Correlated Topic Modelling (CTM) [37], and Non-negative matrix Factorisation (NMF) [38]. BERTopic was chosen for this study due to its ability to compensate for the limitations of other topic modelling methodologies.

LDA is a widely-used traditional methodology for topic modelling. It is a probabilistic generative model that infers topics based on word frequency and models the distribution of words by topic. The model assumes that each document can have multiple topics simultaneously. Since it takes TF-IDF as input and ignores the order of words and relies only on the frequency of occurrence, it can be limited in detecting the relevance between words. Further, the researcher must manually set the number of topics, which can be time-consuming to optimize and leave room for analyst subjectivity. BERTopic uses a class-based TF-IDF to analyse the overall context and automatically determine the optimal number of topics, making it a faster and more efficient method than LDA.

LSA is a method which is easy-to-use and fast, but it is resource-intensive as the analysis must be redone when new values are updated, and its predictive power is reduced when data is outside the normal distribution. CTM is a powerful model that can consider frequency and context simultaneously, but it can be time-consuming to perform and too complex.

NMF is a useful methodology for identifying keywords as it is based on a limited number of topics. However, it may not yield satisfactory results for large datasets. Additionally, NMF employs a linear approach to word representation, which can result in suboptimal word embeddings. On the other hand, BERTopic is effective for large datasets and can use the BERT model to extract topics in a more flexible manner. However, BERTopic utilises BERT's embeddings, which provide rich semantic information.

BERTopic is a methodology that can address the limitations of existing methodologies mentioned above. Previous studies have also shown that BERTopic performs well when compared to other topic modelling methodologies on the same data [24]. Therefore, in this study, BERTopic was used for topic modelling using patent data of customer service technologies.

The data used in this study was crawled using Google Patents. In order to identify trends in customer service technologies over time, this study set the date range of the patent data from January 2000 to December 2022, and used English-language patents filed with the International Patent Organization (WO) to facilitate topic extraction. The keyword customer service was set as the top search term to collect data that meet the research objectives of this study, and detailed keywords of digital transformation and Industry 4.0 technologies were included as sub-search terms based on the literature review. The part of the patent search query expression that this study used to collect the data is as follows: “TI=(customer service) OR AB=(customer service) OR TAC=(data science) OR TAC=(data analytics) OR TAC=(Big data) OR TAC=(business intelligence) OR TAC=(machine learning) OR TAC=(deep learning) OR TAC=(artificial intelligence) OR TAC=(cloud computing) OR TAC=(data mining) OR TAC=(social media) OR TAC=(internet of things) country: WO before priority:20221231 after:priority:20000101 language: ENGLISH type: PATENT”. The initial dataset was constructed by crawling 3207 patent data retrieved by the final search, and after pre-processing to remove errors and data stored in incorrect format, the final 3039 patents were defined as the final dataset for topic modelling.

The crawled data consisted of the following columns: ID, Title, Abstract, Description, Claims, Inventors, Current Assignee, Patent Office, Publication Date, and URL. After converting this data into a data frame, missing values in the ‘Abstract’ and 'Claims' columns were replaced with empty strings and rows with missing values in the ‘Publication Date’ column were deleted. The ‘Abstract’ and 'Claims' columns were then converted to string type, and the ‘Publication Date’ column was converted to datetime type. After processing the missing values and converting the data types, we defined a new data frame consisting of rows with the value of the ‘Publication Date’ column equal to or less than ‘2022-12-31'. The ‘Abstract’ and 'Claims' columns of the new data were combined to create a single text column.

The analysis was conducted in the Google Colab environment. Colab is a cloud-based Jupyter Notebook environment provided by Google that allows developers to write and run Python code in a web browser. It is also connected to Google Drive to store and share notebook files, and has the advantage of using hardware accelerators such as GPUs and TPUs because it is connected to the full Google Cloud platform. As a result, this environment can be used to quickly process large datasets or train complex models while performing machine learning, deep learning, and data analysis. Before starting the analysis, it was necessary to install packages and load several libraries and modules. First, the ‘BERT’ package was installed and several other libraries were imported for data manipulation and analysis, including ‘pandas’ for data manipulation, ‘numpy’ for array operations, and ‘re’ for handling regular expressions to search, split, and replace strings. Additionally, from ‘Scikit-learn’, ‘CountVectorizer’ was imported to transform text data into a matrix of token counts, and ‘ENGLISH_STOP_WORDS' was imported to remove English stop words during text preprocessing. Other modules such as ‘keyBERTInspired’ were imported to extract key terms that enhance the representativeness of topics, and ‘MaximalMarginalRelevance’ was imported to select keywords that reduce redundancy and include diverse topics. Finally, ‘pyplot’, a subpackage of the ‘matplotlib’ library, was imported for data visualization and graph generation.

The process of data cleaning and vectorization kicks off by setting up a CountVectorizer from the sklearn library. This study configured it with the parameter stop_words set to “English”, which helps filter out common English stop words that don't add much value. The CountVectorizer then tokenizes the text documents, breaking them down into individual words and creating a matrix that shows the frequency of each word from a set vocabulary. Then this study compile a regular expression pattern to hunt down and remove specific domain-related words that aren't effective for topic modelling. For instance, the pattern r'\b (claim|method|said|subject)\w*\b' with the re.IGNORECASE flag matches any word starting with “claim”, “method”, “said”, or “subject”, regardless of case. Each document in the dataset is then cleaned to filter out these words. The text is split into words using non-word characters as delimiters (\W+), and any word that matches the pattern is removed. This step is vital for cleaning the text data, as it helps get rid of noise or redundancy that could prevent topic modelling from performing ineffectively. Next, this study set up a ClassTfidfTransformer with reduce_frequent_words set to ‘True’. This transformer tweaks the term weights in the document-term matrix by evaluating how important terms are to specific topics or classes. It gives higher weights to words that are more indicative of a particular topic and lowers the influence of very common words that might not be that informative. In addition, this study initializes the MaximalMarginalRelevance model with a diversity parameter of 0.2. This model generates a variety of keyword representations for each topic, ensuring they are both relevant and diverse. The diversity parameter balances relevance and diversity, and with 0.2, it emphasizes diversity by 20 %. This helps reduce redundancy in the keyword list and offers a wider perspective of the topic's content.

## 3 Analysis results

**Fig. 1:**
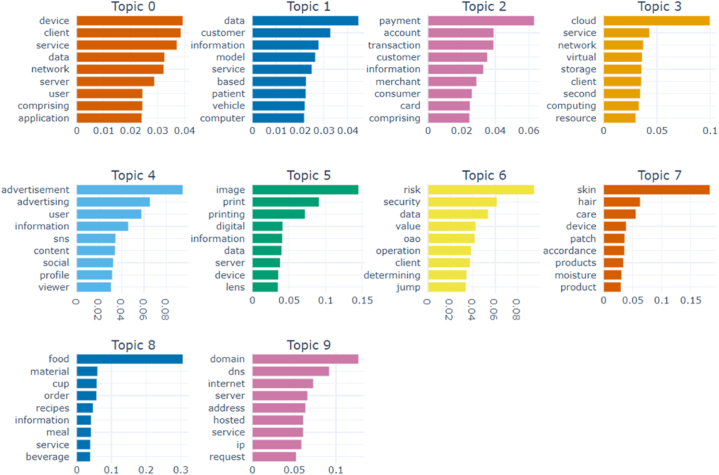
Topic word scores.

After topic modelling with the final dataset, the 10 topics and the top 4 words of each topic are shown in Table 2 and Fig. 1 below.

**Table 2:** Topics and words.

Topic	Number of Documents	Word1	Word2	Word3	Word4
0	613	Device	Client	Service	Data
1	268	Data	Customer	Information	Model
2	216	Payment	Account	Transaction	Customer
3	201	Cloud	Service	Network	Virtual
4	47	Advertisement	Advertising	User	Information
5	35	Image	Print	Printing	Digital
6	30	Risk	Security	Data	Value
7	16	Skin	Hair	Care	Device
8	14	Food	Material	Cup	Order
9	13	Domain	DNS	Internet	Server

This study defined the name of each topic based on the top keywords of each extracted topic and previous studies. Topic 0 is “user-centric network service design”, which has the largest number of patents in Table 2. User-centric network services provide services with low prices, high profitability and added value. As the value of services has changed from production-oriented to customer-oriented, and the Internet of Things technology has emerged since the Fourth Industrial Revolution, research on Topic 0 has been conducted in various fields [[39], [40], [41]].

Topic 1 is “analyse customer and service information using data-based models”. Analysing customers and services has become increasingly important as the amount of data is growing rapidly and the amount of information about services provided by customers is increasing through social media and reviews. In addition, the use of data-based models has become essential for companies to select key customer bases and provide services that can generate consistent profits.

Topic 2 is “identifying customer payments patterns and trends through payment data analysis”. Based on the results of analysing customers' payment data, companies can provide personalized experiences by identifying each customer's purchasing patterns and provide improved services by identifying trends.

Topic 3 is “cloud computing”. Cloud refers to a system where files and information such as documents, photos and music are stored on personal servers on the Internet, and cloud computing refers to a computing environment where IT-related services can be accessed instantly via servers on the Internet. Cloud services allow businesses to reduce costs and increase flexibility of access, while improving security. Cloud computing is one of the main technologies of digital transformation and is being applied in various industries using artificial intelligence [42] and blockchain technology [[43], [44], [45]], especially in the financial sector [46].

Topic 4 is “recommending advertisements based on user's profile on social network services”. Recently, social media users' profiles, algorithms and advertisement recommendations are increasing, and the top keywords of Topic 4 are advertisement and advertising, which is judged to be due to the inclusion of words such as information, social, or viewer as keywords.

Topic 5 is “processing and analysis of digital image data”, and the types of services using digital image data include services using facial recognition technology, product and image search functions [47]. Topic 6 is “data security and valuation”. With the growth of services using large amounts of data, the importance of data security and privacy is steadily increasing.

Topic 7 and 8 are “cosmetics recommendation system” and “customised meal and nutrition service”, respectively. Both topics are characterised by recommendation services for specific areas and customised services. The reason why the topics were identified as recommendations and customised services for specific fields is that the top four keywords in Topic 7 were skin, hair, care, and device, and the top four keywords in Topic 8 were food, material, cup, and order.

Topic 9 is “monitoring user requests using Internet servers hosed on domain name system (DNS)”. DNS is a TCP/IP network service that translates a domain or host name into a numeric IP address. This technology provides the ability to monitor customer requests in real time, allowing a company's customer service department to quickly respond to user requests and resolve issues.

**Fig. 2:**
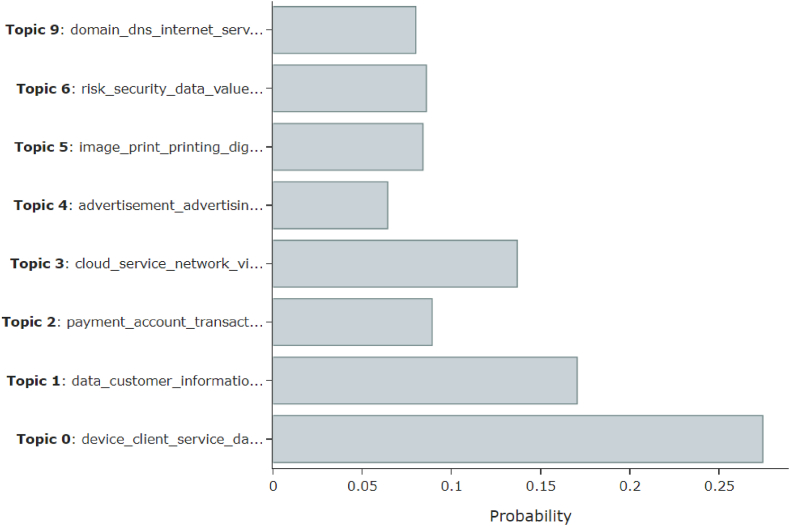
Topic probability distribution.

For each of the 10 extracted topics, this study examined how much each patent is related to the topic in Fig. 2. The larger the value of probability, the stronger the correlation between the topic and the patent, which indicates the importance of the topic. Thus, the top three most important topics are Topic 0, Topic 1, and Topic 3. On the other hand, Topics 7 and 8 were excluded from the comparison graph because their probabilities were less then 0.01, indicating that they have very low relative importance.

**Fig. 3:**
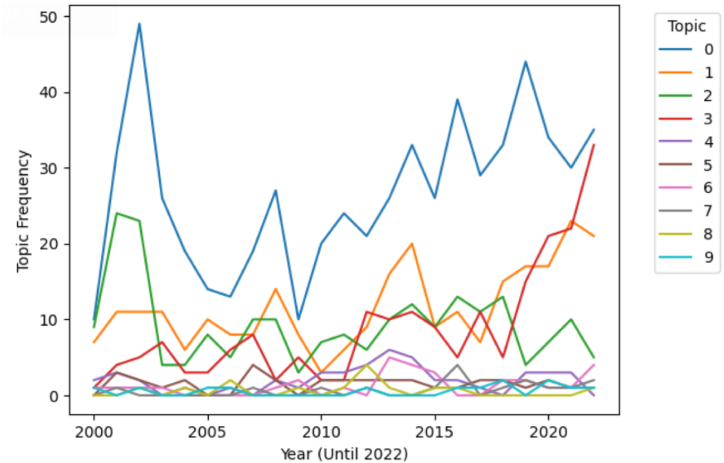
Topic distribution by year.

Fig. 3 shows the annual trend of patents by topic, analysed from 2000 to 2022. As of 2022, the top four topics are Topic 0, Topic 1, Topic 2, and Topic 3. Topic 0 has fluctuated since its peak in the early 2000s and is expected to increase from 2022 onwards. As user-centric network services have evolved along with network technology, it can be said that related services have also evolved since the late 1990s and early 2000s, when the Internet rapidly spread and became commercialised. Topic 2 showed a similar trend to Topic 0 until the mid to late 2010s, but by the 2020s it showed the opposite trend to Topic 0, and as of 2022 it shows a declining trend. The reason for the recent decline in Topic 2 is that the related technology has already grown considerably and numerous patents have been filed. However, as more advanced related technologies emerge in Topic 2, it is expected to resume its upward trend. In the case of Topic 1, it was not a noteworthy topic until 2010, but since the 2010s it has been increasing and decreasing. As of 2022, it shows a declining trend but it seems to be a topic worth monitoring in the long run.

From the results, the most noteworthy topic is Topic 3, which has shown a sharp increase since the late 2010s, when the technologies of the Industrial 4.0 began to be commercialised in earnest. As of 2022, it is on par with Topic 0 and has potential to surpass Topic 0 considering the time series trend. The trend of Topic 3 from the late 2010s to 2022 shows a strong increase, followed by a pause around 2020 and then a sharp increase again. Considering that COVID-19 affected the word from 2020 to 2021, the increase in the late 2010s is likely due to the impact of the Industrial 4.0, and the increase in the 2020s is likely due to the acceleration of digital transformation due to COVID-19, which has affected the field of “untact” customer service [48].

## 4 Conclusion

The perceived value of customer service is constantly changing over time. Therefore, as the main value of the service changes, the main technology changes accordingly, so it is necessary to analyse the main technology in the field of customer service and identify patterns. In the process of literature review, there are no studies that performed topic modelling in the field of customer service, and since BERTopic is a recently emerged technology, there are not many studies that utilised it. This study differs from previous studies in that it used BERTopic to analyse patent data in the field of customer service using a pre-trained large language model to define each extracted topic as a technology and derive patterns accordingly for a large amount of patent data.

By conducting topic modelling using patents in the customer service industry from January to December 2022, this study contributes to identifying trends at a specific point in time by defining major technologies as topics and comparing the changes in the frequency of each topic over time and the resulting trends. In addition, it is possible to check the trend of which topics increase in frequency before and after certain events, such as the emergence of the Fourth Industrial Revolution, which has greatly affected the customer service field, and the full-scale and acceleration of digital transformation, and draw patterns as a result. Therefore, this study identifies the time series trend of patents in the customer service field and suggests the effectiveness of predicting future trends in technology through topic modelling. This study found that the most frequent topic in 202 is user-centric network service design, and the topic with the most significant increase is cloud computing. User-centric network service design has been identified as a key technology in customer service as the value of customer service shifts to a more consumer-centric form. Cloud computing has been identified as one of the key technologies being developed most intensively in 2023 for the digital transformation of customer service.

However, this study has some limitations: lack of diversity and lack of observations. The first is the lack of diversity in that this study only used patent data for textual analysis. Patent data is a useful source for identifying technological development trends and detailed focus technologies, but patent data cannot explain the entire customer service field. For example, it is possible that what may be recognised as a key technology in the customer service industry has not yet been patented, or may be introduced or proposed through academic papers or reports rather than patents.

Secondly, in this study, a total of 3039 patents were used for analysis, but as shown in previous studies, when using social media or reviews as data for analysis, more than 10,000 data are usually used. To search for patents in the customer service industry and technology, this study used the keywords of specific industries and technologies that are highly relevant to the customer service industry, as well as the keyword “customer service” in the patent search queries. While there is no clear minimum number of texts or documents required for topic modelling using large language models, it is possible that future research could uncover other notable trends if the search terms were more inclusive of customer service analogues or related technologies and industries. However, this would presuppose that the keywords have a clear relevance to the customer service industry.

Lastly, this study used HDBSCAN to cluster representations of documents and create topics based on the similarities between clusters and finally determine the number of topics. HDBSCAN is a density-based clustering algorithm that works by grouping dense regions of data into clusters. Internally, HDBSCAN automatically selects the appropriate number of topics by utilizing a density-based algorithm to determine the optimal number of clusters. However, when performing topic modelling, the algorithm does not necessarily automatically determine the optimal number of topics. It is also possible for the analyst to force an arbitrary number of topics to be fixed. For example, patent documents in the field of customer service could be topic modelled with 15, 20, 30, etc. topics. For the sake of clarity, this study has included the keyword extraction results for each of these numbers in the Appendix Section. In this case, more detailed technical fields can be identified, and topics with fewer documents but closely related to promising future technologies can also be identified. Therefore, future research may consider an approach to determine the appropriate number of topics by considering various numbers of topics and comparing the modelling results of each to determine the appropriate number of topics. However, forcing an excessive number of topics may result in unclear technology flows or difficulty in interpreting the topic modelling results.

## Data availability statement

Data will be made available on request.

## CRediT authorship contribution statement

**Chaeyeon Kim:** Writing – original draft, Methodology, Formal analysis, Data curation. **Juyong Lee:** Writing – review & editing, Writing – original draft, Validation, Supervision, Investigation, Conceptualization.

## Declaration of competing interest

The authors declare that they have no known competing financial interests or personal relationships that could have appeared to influence the work reported in this paper.

## Figures and Tables

**Table A1:** Topics and words (set the number of topics to 15).

Topic	Number of Documents	Keywords
0	980	data, service, information, user, client, development
1	840	client, service, device, data, network, server
2	360	customer, payment, information, transaction, system
3	52	patient, medical, health, data, information
4	26	nf, nwdaf, function, val, response, message
5	22	risk, data, oao, value, operation, jump, fraud
6	18	print, printing, image, film, postage, editing
7	17	image, digital, 2 d, images, search, code, photo
8	17	search, query, bot, result, user, corpora, interaction
9	15	skin, hair, care, patch, accordance, products
10	15	food, material, vending, cup, order, infusion
11	14	flow, traffic, bandwidth, rate, network, uplink
12	13	appointment, scheduling, task, seating, temporary
13	12	domain, dns, internet, address, ip, server
14	11	content, peer, rights, digital, provider, identity

**Table A2:** Topics and words (set the number of topics to 20).

Topic	Number of Documents	Keywords
0	1156	data, service, information, client, network
1	222	payment, transaction, account, customer, information
2	158	service, data, client, web, application, request
3	105	authentication, client, device, user, key, cloud
4	86	customer, model, data, vector, learning, machine
5	85	vehicle, power, energy, information, data, access
6	85	wireless, mobile, location, device, client, network
7	83	cloud, virtual, storage, service, network, computing
8	81	video, media, content, device, digital, client
9	57	patient, medical, health, data, support, information
10	57	voice, telephone, communications, network, line
11	44	image, print, printing, digital, images, service
12	39	nf, traffic, service, nwdaf, network, flow, virtualization
13	33	advertising, viewer, electronic, content, display
14	26	risk, security, oao, data, operation, value
15	19	optical, downlink, line, fiber, electrical, unit
16	18	skin, hair, color, care, music, patch, accordance
17	17	sns, user, information, social, networking, login
18	15	food, material, cup, order, infusion, ingredients
19	14	appointment, scheduling, seating, callback, manage

**Table A3:** Topics and words (set the number of topics to 30).

Topic	Number of Documents	Keywords
0	1098	data, service, information, client, device
1	198	cloud, virtual, storage, computing, service
2	115	service, business, client, application, data
3	96	video, media, content, stream, device, digital
4	92	authentication, client, device, security, service
5	88	payment, account, transaction, card, merchant
6	84	product, information, purchase, customer, coupon
7	71	model, learning, vector, customer, machine, data
8	68	mobile, wireless, location, device, data, client
9	55	power, energy, building, maintenance, management
10	51	patient, medical, health, data, support
11	39	telephone, network, voice, atm, subscriber
12	38	order, inventory, customer, delivery, menu
13	32	advertisement, advertising, viewer, means, content
14	26	nf, nwdaf, val, function, producer, mcdata, rest
15	25	customer, interaction, parameters, marketing, electronic
16	23	optical, line, downlink, fiber, electrical
17	20	vehicle, car, parking, rental, transponder
18	20	search, query, bot, result, user, corpora
19	18	risk, oao, value, jump, operation, fraud
20	18	image, lens, content, digital, images, search
21	17	print, printing, image, 2 d, editing, postage
22	17	skin, hair, care, patch, accordance, moisture
23	16	subscription, resource, directory, monitoring, request
24	14	domain, dns, internet, hosted, address, ip
25	14	sns, user, social, information, living, network
26	14	food, material, cup, infusion, order, ingredient
27	12	scheduling, appointment, task, seating, temporary
28	12	gaming, game, wager, event, handling, gameplay
29	11	voice, speech, semantic, data, audio, text
